# Pediatric Fibrinogen PART II—Overview of Indications for Fibrinogen Use in Critically Ill Children

**DOI:** 10.3389/fped.2021.647680

**Published:** 2021-04-21

**Authors:** Gemma Louise Crighton, Elise J. Huisman

**Affiliations:** ^1^Department of Hematology, Royal Children's Hospital, Melbourne, VIC, Australia; ^2^Department of Hematology, Erasmus MC–Sophia Children's Hospital, Rotterdam, Netherlands; ^3^Department of Clinical Chemistry and Blood Transfusion, Erasmus MC, Rotterdam, Netherlands; ^4^Department of Transfusion Medicine, Sanquin Blood Supply, Amsterdam, Netherlands

**Keywords:** fibrinogen, children, hypofibrinogenemia, cryoprecipitate, fibrinogen concentrate, bleeding, hyperfibrinolysis

## Abstract

Bleeding is frequently seen in critically ill children and is associated with increased morbidity and mortality. Fibrinogen is an essential coagulation factor for hemostasis and hypofibrinogenemia is an important risk factor for bleeding in pediatric and adult settings. Cryoprecipitate and fibrinogen concentrate are often given to critically ill children to prevent bleeding and improve fibrinogen levels, especially in the setting of surgery, trauma, leukemia, disseminated intravascular coagulopathy, and liver failure. The theoretical benefit of fibrinogen supplementation to treat hypofibrinogenemia appears obvious, yet the evidence to support fibrinogen supplementation in children is sparce and clinical indications are poorly defined. In addition, it is unknown what the optimal fibrinogen replacement product is in children and neonates or what the targets of treatment should be. As a result, there is considerable variability in practice. In this article we will review the current pediatric and applicable adult literature with regard to the use of fibrinogen replacement in different pediatric critical care contexts. We will discuss the clinical indications for fibrinogen supplementation in critically ill children and the evidence to support their use. We summarize by highlighting current knowledge gaps and areas for future research.

## Introduction

### Bleeding in Critically Ill Children

Bleeding is a common complication observed in critically ill children and appears to be multifactorial (1). In children admitted to the pediatric intensive care unit (PICU), clinically significant bleeding is reported to occur in ~10% with higher rates reported in those receiving mechanical circulatory support (1–3). Bleeding is associated with adverse outcomes including longer PICU stays, increased vasopressor support, increased red cell transfusions (1, 2) and in children requiring extracorporeal membrane oxygenation (ECMO) increased mortality (3).

### Hypofibrinogenemia in Critically Ill Children

Fibrinogen (Factor I) is an essential hemostatic protein, with a key role in all aspects of normal hemostasis (4, 5). In critically ill children, fibrinogen has not been identified as an independent risk factor for bleeding. Studies looking at risk factors for bleeding in critically ill children have either not evaluated fibrinogen levels (1) or found the opposite, namely that fibrinogen levels are higher in critically ill children with bleeding (2). This finding most likely reflects that fibrinogen is an acute phase reactant and therefore may be elevated in the critical care context, in the context of sepsis, infection, or inflammation (6). Hypofibrinogenemia has been identified as an important risk factor for bleeding in other pediatric and adult settings (7–9). It is therefore important to appreciate a low fibrinogen level and recognize its potential contribution to bleeding.

See Pediatric Fibrinogen Part I—Pitfalls in Fibrinogen Evaluation and Use of Fibrinogen Replacement Products for further information about the diagnostic tools used to measure fibrinogen in critically ill children and the available fibrinogen replacement products.

### Hypofibrinogenemia in Critically Ill Children

**Figure 1:**
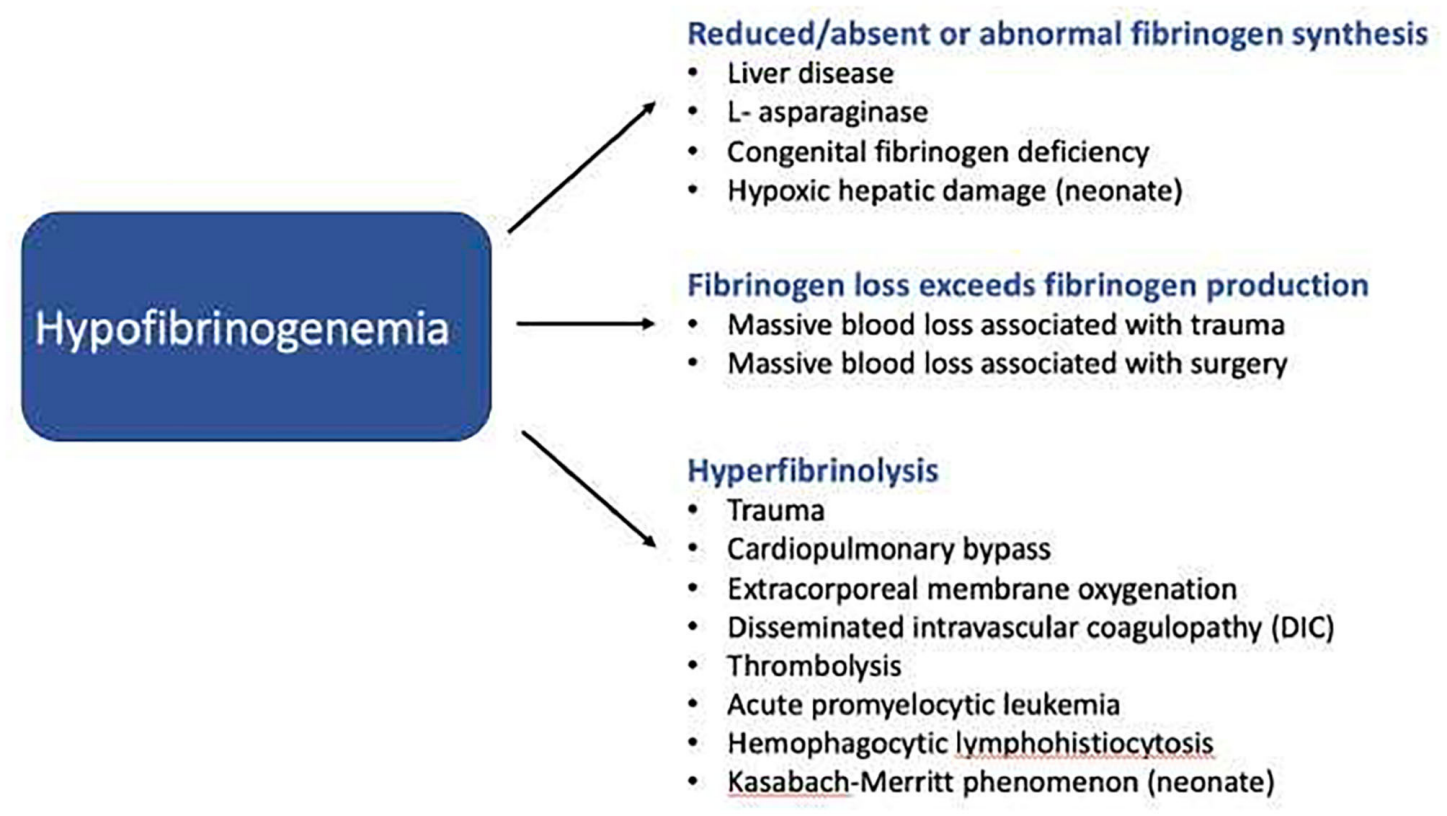
Machanisms by which hypofibrinogenemia may develop in critically ill children.

Hypofibrinogenemia in the PICU is most commonly acquired, or secondary to an underlying process. See Figure 1. It may result from:

A) ***Reduced/absent or abnormal fibrinogen synthesis*** as seen in severe liver disease (10) or, in the rare instance of congenital fibrinogen disorders.
B) ***Fibrinogen loss exceeding fibrinogen production*** for example, during massive blood loss (11).
C) ***Hyperfibrinolysis***, a condition where fibrinolytic activity exceeds fibrin formation, which may be observed in trauma (12), post cardiopulmonary bypass (CPB), with extracorporeal membrane oxygenation (ECMO) (9, 13) and in disseminated intravascular coagulopathy (DIC) (14). See Figure 1.

Cryoprecipitate and fibrinogen concentrate are most commonly used to provide fibrinogen supplementation, and both effectively restore fibrinogen levels (15–18). They may be given to provide fibrinogen replacement during active bleeding or as prophylaxis to prevent bleeding. A prospective study of cryoprecipitate use in England, found that most cryoprecipitate given to children was for prophylactic, rather than therapeutic indications, specifically in the context of pediatric cardiac surgery, critical care, or the hematology/oncology setting (19).

## The Critical Fibrinogen Level

There are a number of the age-related differences in the hemostatic systems including fibrinogen and fibrinolysis between neonates and older children and adults (20, 21). The critical fibrinogen threshold level and the risk of bleeding is debated in children of all ages.

Nevertheless, it is obvious that there is a strong association between fibrinogen level and bleeding severity in individuals with congenital fibrinogen deficiency, with bleeding rarely reported at fibrinogen levels >100 mg/dL (22). The International Council for Standardization in Hematology considers a fibrinogen level of 50–200 mg/dL to be a critical test result but recognizes the overlap with normal references ranges; a result of 150 mg/dL, may be normal or critical depending on the clinical context (23). In the adult surgical setting a fibrinogen level of <150–200 mg/dL, increases the risk of perioperative and postoperative bleeding tendency (7). Similarly in children undergoing cardiac surgery, post-CPB fibrinogen levels of <150 mg/dL have been shown in both retrospective (9), and prospective (8) studies to be associated with increased rates of post-operative bleeding.

As a result, international clinical guidelines outlining pediatric fibrinogen replacement guidance also, having varying fibrinogen thresholds and targets. See Table 1.

*In summary, fibrinogen is a key hemostatic protein and hypofibrinogenemia is an important risk factor for bleeding, but the critical threshold is debated. Fibrinogen levels need to be interpreted in their clinical context, e.g., a child's age, the presence of inflammation or active bleeding and any planned surgery*.

**Table 1:** Guidance for pediatric fibrinogen replacement therapy from international guidelines.

**Guideline**	**Type of guideline and patient population**	**Transfusion guidance and indications**	**Dosing information**
**Cryoprecipitate**			
BSH (1)	Neonatal and pediatric specific transfusion guideline	*Recommendations* •Prophylactic cryoprecipitate should not be routinely administered to non-bleeding children with decreased fibrinogen including prior to surgery •Cryoprecipitate should not be used for congenital hypofibrinogenemia unless fibrinogen concentrate is unavailable •Cryoprecipitate may be considered for fibrinogen <100 mg/dL for surgery at risk of significant bleeding or to critical sites •In the setting of DIC—cryoprecipitate may be given if fibrinogen is <100 mg/dL despite FFP, or in conjunction with FFP for very low or rapidly falling fibrinogen •Clinically significant bleeding following CPB and fibrinogen <150 mg/dL, specific component replacement may be warranted Practice Point In patients with critical bleeding—early use of FFP, platelets and cryoprecipitate is recommended in order to reduce coagulopathy and thrombocytopenia	*Cryoprecipitate* 5–10 mL/kg with higher volumes in bleeding patients
		Guidance Massive blood loss aim fibrinogen >150 mg/dL	Massive blood loss *Cryoprecipitate* 10 mL/kg
NBA (2)	Neonatal and pediatric specific PBM guideline	Practice Point •In children, the decision to transfuse cryoprecipitate or fibrinogen concentrate should take into account the potential risks and benefits. The decision should be based not only on laboratory investigations but also on assessment of the patient's clinical condition. Factors that may influence the decision include active bleeding, medications affecting coagulation status, and congenital and acquired bleeding disorders	
		Expert opinion point •Cryoprecipitate should be used to treat active bleeding when the fibrinogen level is <150 mg/dL. •A target level of 200 mg/dL may be appropriate in certain situations (e.g., critical bleeding is occurring or anticipated)	*Cryoprecipitate* 5 mL/kg
Italian neonatal transfusion guideline, 2015 (3)	Neonatal transfusion guideline	Recommended interventions •Term and preterm neonates with hypofibrinogenemia but no bleeding, observation is recommended	
		Recommended interventions •Term and preterm neonates with hypofibrinogenemia and bleeding or about to undergo an invasive procedure, cryoprecipitate is recommended	*Cryoprecipitate* 5–10 mL/kg
SABM (4)	Pediatric PBM Standard in PBM Guideline	Indicators •The decision to transfuse cryoprecipitate should be based on laboratory studies, including point-of-care viscoelastic testing if available, fibrinogen concentration, the patient's clinical status and the etiology of the patient's coagulopathy •Fibrinogen concentrate may alternatively be considered	*Cryoprecipitate* Volume should be calculated based on weight and desired increase in fibrinogen concentration and improvement in coagulations indices
Faraoni, NATA (5)	Pediatric cardiac surgery guideline	Recommendations •The authors suggest FFP should be considered for treating hypofibrinogenemia in bleeding neonates and children **only** when cryoprecipitate or fibrinogen concentrates are not available •In bleeding neonates and children, the authors recommend hypofibrinogenemia diagnosed either by Clauss method (<150 mg/dL) or viscoelastic tests (based on institution-specific algorithm) should be treated either with cryoprecipitate or fibrinogen concentrate	
NICE guidelines 2015 (6)	Guideline for adults, young people, and children over 1 year	Generic Recommendations •Do not offer cryoprecipitate transfusions to correct the fibrinogen level in patients who: are not bleeding and are not having invasive procedures or surgery with a risk of clinically significant bleeding	*Cryoprecipitate* 5–10 mL/kg up to a maximum of 2 pools
		•Consider cryoprecipitate transfusions for patients with major hemorrhage who have clinically significant bleeding **and** a fibrinogen level <150 mg/dL •Consider prophylactic cryoprecipitate transfusions for patients with a fibrinogen level <100 mg/dL who are having invasive procedures or surgery with a risk of clinically significant bleeding	
Blood Easy 4, Canada, 2016 (10)	Blood Transfusion Guideline	Generic Indications •For bleeding with fibrinogen <100 mg/dL •For massive hemorrhage with fibrinogen <150–200 mg/dL •For acute phase of APML with fibrinogen <150 mg/dL •ICH secondary to treatment with tPA with fibrinogen <200 mg/dL •Treatment of bleeding in patients with vWD or Hemophilia A, **only** when: Factor concentrates are unavailable, remote geographic region and DDAVP is unavailable or ineffective	*Cryoprecipitate* •1 unit/10 kg body weight to a maximum of 10 units •(~4000 mg fibrinogen)
**Fibrinogen concentrate**			
German Medical Association, 2014 (7)	Blood Transfusion Guideline with guidance for adults and children.	Recommendations—Acquired fibrinogen deficiency •Fibrinogen can be substituted perioperatively in interventions or lesions with the risk of acute bleeding and confirmed fibrinogen deficiency (massive transfusion, dilution and loss coagulopathy) •Fibrinogen can be substituted in synthesis disorders (liver damage) with fibrinogen deficiency or in hemorrhagic dysfibrinogenemias as prophylaxis and therapy of hemorrhage and confirmed fibrinogen deficiency •Fibrinogen can be substituted as a prophylaxis and therapy of hemorrhage and confirmed fibrinogen deficiency of different origin (e.g., acute leukemia, asparaginase therapy, obstetrical complications, postoperatively) *General Recommendations—Acquired fibrinogen deficiency* •The critical threshold values for the occurrence of spontaneous bleeding are <100 mg/dL (in severe hemorrhage 150 mg/dL) •The fibrinogen level should always be specifically determined. An indirect determination using PT or APTT is not sufficient for any decisions regarding substitution therapy. The detection limit of the laboratory assay must be taken into account •Following administration, fibrinogen levels should be monitored and maintained above the critical threshold value (~100 mg/dL)	*Fibrinogen concentrate* Mean adult dose in acquired fibrinogen deficiency 3,000–5,000 g
European trauma guideline, 2019 (8)	Adult trauma guideline^*^	Generic Recommendations *Initial coagulation resuscitation* •In the initial management of patients with expected massive hemorrhage, we recommend one of the two following strategies: •FFP or pathogen-inactivated FFP in a FFP:RBC ratio of at least 1:2 as needed. •Fibrinogen concentrate and red cells *Fibrinogen supplementation* We recommend treatment with fibrinogen concentrate or cryoprecipitate if major bleeding is accompanied by hypofibrinogenemia (viscoelastic signs of a functional fibrinogen deficit or a plasma Clauss fibrinogen level ≤ 150 mg/dL) *Coagulation factor concentrate-based management* •If a CFC-based strategy is used, we recommend treatment with factor concentrates based on standard laboratory coagulation parameters and/or viscoelastic evidence of a functional coagulation factor deficiency •We suggest that monitoring of FXIII be included in coagulation support algorithms and that FXIII be supplemented in bleeding patients with a functional FXIII deficiency	*Fibrinogen concentrate* Adult dose 3,000–4,000 g
ESA 2017 (9)	Perioperative guideline with pediatric surgery section	No specific recommendation made for fibrinogen replacement in pediatric surgery, as neither the optimal threshold for initiation of fibrinogen replacement nor the dose required to reach the targeted fibrinogen concentration, have been proven by high-quality data	

* *Do not provide specific recommendations for children, but state that children who have not been pre-treated with anticoagulant or antiplatelet agents should generally be managed in the same manner as the normal adult patient*.

*APML, acute promyelocytic leukemia; APTT, activated partial thromboplastin time; CPB, cardiopulmonary bypass; CFC, clotting factor concentrate; DDAVP, de-amino-D-arginine vasopressin; DIC, Disseminated intravascular coagulopathy; FFP, fresh frozen plasma; ICH, intracranial hemorrhage; FVIII, Factor VIII; FXIII, Factor XIII; PBM, Patient blood management; PCC, Prothrombin complex concentrate; RBC, red blood cells; PT, Prothrombin Time; tPA, tissue plasminogen activator; vWD, von Willebrand disease; vWF, von Willebrand factor*.

*AABB, American Association of Blood Banks; BSH, British Society of Hematology; ESA, European Society of Anesthesiology; NATA, Network for the Advancement of Patient Blood Management, Hemostasis, and Thrombosis; NBA, National Blood Authority; NICE, National Institute for Health and Care Excellence; SABM, Society for the Advancement of Blood Management*.

## The Evidence Base

### Systematic Reviews and Randomized Controlled Trials

There is a lack of high-quality evidence to support the prophylactic or therapeutic use of cryoprecipitate and fibrinogen concentrate in children (24–28).

Existing evidence comes from the 2018 Cochrane systematic review, which evaluated the efficacy and safety of fibrinogen concentrate, with respect to the primary outcomes of mortality and thromboembolic events, in addition to secondary bleeding outcomes (27). The review identified 22 randomized controlled trials (RCT)s (*n* = 1,535) looking at fibrinogen concentrate of which three were pediatric (*n* = 143) (27). On review of the trial settings, 15 of the 22 trials were conducted in the elective perioperative setting, three in the trauma setting, two in women with post-partum hemorrhage and one in the context of liver transplantation (27). Ten of the 22 trials looked at prophylactic fibrinogen indications, nine compared fibrinogen concentrate with placebo and one trial evaluated two different fibrinogen concentrate intervention points based on FIBTEM. The remaining 12 trials were therapeutic trials, eight compared fibrinogen concentrate with placebo, and four compared fibrinogen concentrate to an active comparator (fresh frozen plasma [FFP], cryoprecipitate, or platelets) (27). The systematic review was significantly limited by the quality of the data published; trials were conducted in heterogeneous clinical settings, with varying interventions, primary outcomes, and different time points for outcome analysis (27). Therefore, meta-analysis could not be performed. Study samples sizes were small (mean 70 participants [range 20–249]) and none of the studies were powered to detect harm or adverse events, limiting their generalizability to clinical practice. Nevertheless, the systematic review observed that fibrinogen supplementation may reduce a patient's risk of receiving a red cell transfusion when compared with placebo in both cardiac and non-cardiac settings. In addition, prophylactic fibrinogen concentrate may reduce post-operative bleeding (27).

Two other recent systematic reviews have evaluated fibrinogen replacement therapies and included pediatric studies (26, 28). The first systematic review evaluated the use of fibrinogen concentrate in perioperative settings and identified 21 RCTs (*n* = 1,605) where fibrinogen concentrate was given either pre-emptively to prevent bleeding or to treat intraoperative bleeding (28). Two identified studies were performed in children (*n* = 114) (17, 29). Overall there was considerable variability in the studies with respect to the patient populations, study designs, comparative arms, and outcomes, including bleeding rates. Only seven of the 12 trials that used fibrinogen concentrate to treat clinically relevant bleeding reported decreased bleeding tendency (28). The study authors rationalized that different study designs might explain the inconsistent results. They advocated for better-designed trials that evaluated patients with significant bleeding and hypofibrinogenemia (28).

The second systematic review evaluated the efficacy and safety of cryoprecipitate compared with fibrinogen in bleeding patients and identified one RCT (*n* = 63) conducted in children undergoing cardiac surgery and three observational studies that included adults. All studies were at high risk of bias and the authors concluded that it was not possible from the current evidence to recommend one fibrinogen replacement product over another (26).

From the systematic reviews and literature search, eight published pediatric RCTs (*n* = 616) (17, 18, 29–33) evaluating fibrinogen replacement in the surgical setting were identified for the purpose of this review. Of the eight RCTs, five are performed in the cardiac surgery setting (17, 18, 32–34), one in the adolescent scoliosis setting (30), one in infants undergoing craniofacial surgery (31), and the final study included both infants undergoing craniofacial surgery and adolescents undergoing scoliosis surgery (29). The details and results will be discussed further in the respective surgical settings and are presented in Table 2.

**Table 2:** Randomized controlled trials of fibrinogen replacement in pediatrics.

**References**	**Setting**	**Population**	**Intervention**	**Comparator**	**Fibrinogen replacement**	**Outcomes**
**Cardiac surgery**						
Cui et al. (33)	Single center—China Not blinded	Children with cyanotic heart disease undergoing cardiac surgery *N* = 40 (only 31 analyzed)	Fibrinogen concentrate in addition to traditional transfusion, guided by TEG (500–1,000 mg)	Traditional transfusion guided by clinical experience	500–1,000 mg (Fibrinogen concentrate type not specified)	FC and TEG guided transfusion (*n =* 17) vs. Standard transfusion (non-TEG guided) (*n =* 14) ^*^Closure time (min): 125 (90–162.5) vs. 138.5 (81.3–194) Total red cell use (U): 0 (0–1.5) vs. 1.5 (0–2.3), *p =* 0.109 Total Plt use (U): 1 (1–1) vs. 1 (0.7–1.9), *p =* 0.984, Total FFP use (mL/kg): 56.6 ± 17.0 vs. 82.5 ± 31.3, *p =* 0.006 FFP ICU first 24 h. (mL/kg): 10.6 ± :6.5 vs. 22.5 ± 13.1 ml, *p =* 0.006 ICU stay (h.): 137 (106.7–161.2) vs. 173.1 (137.7–477.2), *p =* 0.009 Hospital stay (d.): 21 (15.5–30) vs. 32 (24.3–40.3), *p =* 0.006
Galas et al. (17)	Single center–Brazil Outcome assessors blinded	Children (<7 years) undergoing cardiac surgery and diffuse bleeding after CPB and fibrinogen level <100 mg/dL *N* = 63	Fibrinogen concentrate (60 mg/kg)	Cryoprecipitate (10 ml/kg)	Haemocomplettan P ® CSL Behring, Germany	FC (*n =* 30) vs. Cryoprecipitate (*n =* 63) ^*^Median 48 h. blood loss (ml): 320 (157–750) vs. 410 (215–510), ***p=*** **0.672** Post-operative red cell transfusion: 25/30 vs. 32/33, *p =* 0.094 Post-operative FFP: 3/30 vs. 8/33, *p =* 0.137 Post-operative Plt: 0/30 vs. 3/33, *p =* 0.24 Post-operative Cryo: 13/30 vs. 14/33, *p =* 0.942 ICU stay (d.): 10 (6–25) vs. 10 (5–24), *p =* 0.930 Hospital stay (d.): 21 (12–32) vs. 20 (10–38), *p =* 0.895 Death: 0 vs. 0
Massoumi et al. (34)	Single-center–Iran Not blinded	Infants (<2 years) undergoing cardiac surgery *N* = 90	Fibrinogen concentrate (70 mg/kg)	Fresh frozen plasma (10 mL/kg)	Haemocomplettan® P, CSL Behring, Germany	FC (*n =* 45) vs. FFP (*n =* 45)**Chest tube drainage (ml/kg/hr) at 3 h.: 4.77** **±** **2.05 vs. 6.94** **±** **6.05**, ***p=*** **0.02** **Chest tube drainage (ml/kg/hr) at 6 h.: 4.13** **±** **1.84 vs. 6.31** **±** **6.22**, ***p=*** **0.02** **Chest tube drainage (ml/kg/hr) at 12 h.: 3.40** **±** **1.29 vs. 4.52** **±** **4.37**, ***p=*** **0.04** **Chest tube drainage (ml/kg/hr) at 24 h.: 1.93** **±** **6.63 vs. 2.64** **±** **2.18**, ***p=*** **0.04** ICU stay (d.): 3.04 ± 1.79 vs. 3.66 ± 1.80, *p =* 0.89 Total red cells (U): 5(11.1) vs. 9(20), *p =* 0.49 Total Plt (U): 0 (0) vs. 2 (4.44), *p =* 0.38
Downey et al. (18)	Two centers USA Not blinded	Infants (<12 months) undergoing cardiac surgery post CPB protocol *N* = 59	Fibrinogen concentrate (dose to target 300 mg/dL)	Cryoprecipitate (2 units)	RiaSTAP®, CSL Behring, Germany	FC (*n =* 30) vs. Cryoprecipitate (*n =* 29) —ITT analysis ^*^Total all blood product (U): 4 (3–5) vs. 5.5 (4–7), ***p=*** **0.007** Total red cells (U): 2 (1–2.8) vs. 2 (1–2), *p =* 0.383 Total FFP (U): 1 (1–1) vs. 1 (0–1), *p =* 0.263 Total Plt (U): 1 (1–1) vs. 1 (1–1), *p =* 0.257 Total Cryo (U): 0 (0–0) vs. 2 (2–2), *p < *0.001
						FC (*n =* 29) vs. Cryoprecipitate (*n =* 25) —PP analysis Chest tube output (mL/kg): 16.1 (12.6–25) vs. 18.1 (10.9–26), *p =* 0.671 ICU stay (d.): 3 (2–7) vs. 4.5 (2–6), *p =* 0.487 Hospital stay (d.): 7 (4–11) vs. 8 (5–19), *p =* 0.342 Death within 30 day: 1 vs. 0
Siemens et al. (32)	Single-centre UK Partially blinded Trial team not blinded, treating clinicians and patients blinded	Infants (2.5–12 kg) undergoing cardiopulmonary bypass surgery *N* = 111 (monitor cohort, *n =* 21; FC, *n =* 60, placebo, *n =* 30)	Fibrinogen concentrate (individualized dose targeted to FIBTEM-MCF of 8–13 mm)	Placebo (0.9% sodium chloride)	RiaSTAP®, CSL Behring, Germany	FC (*n =* 60) vs. Placebo (*n =* 30) ^*^FibTEM—MCF post dose (mm): 13.0 (3.2) vs. 4.5 (1.7) ^*^Fibrinogen level post dose (g/L): 1.7 (0.4) vs. 0.7 (0.2) 24 h mediastinal drain loss (ml/kg): 11.6 (5.2) vs. 17.1 (14.3), *p =* 0.02 Perioperative red cells, *n* (%): 21 (35%) vs. 12 (40%) Perioperative FFP *n* (%): 3 (5%) vs. 5 (16.7%) Perioperative Plt *n* (%): 8 (13.3%) vs. 5 (16.7%) Perioperative Cryo *n* (%): 5 (8.3%) vs. 2 (6.7%) Thrombosis: 10 vs. 2
**Craniosynostosis surgery**						
Haas et al. (29)	Single center—Switzerland Staff in PICU blinded	Children (median age 10 months) undergoing craniosynostosis surgery *N* = 30	Early Fibrinogen replacement FIBTEM MCF <13 mm	Conventional Fibrinogen replacement FIBTEM MCF <8 mm	Haemocomplettan P® CSL Behring, Germany (30 mg/kg)^#^	Early (*n =* 13) vs. conventional (*n =* 17) fibrinogen replacement ^*^Total red cells in 24 h. (ml/kg): 28.2 (21.2–49.9) vs. 55.5(27.5–61.8), ***p=*** **0.03** Total FFP in 24 h. (mL/kg): 0 vs. 0, *p =* 0.97 Total Plt in 24 h. (mL/kg): 0 vs. 0, *p =* 0.43 Calculated blood loss (%): 90 (78–113) vs. 157 (111–187), *p =* 0.02 ICU stay (d.): 1 (1–1) vs. 1 (1–1), *p =* 0.59 Hospital stay (d.): 9 (9–9) vs. 9 (9–9), *p =* 0.54
Machotta et al. (31)	Single center Netherlands Blinded	Infants and children (5–25 months) undergoing craniosynostosis surgery *N* = 114 (111 in final analysis)	Fibrinogen concentrate (dose to target 300 mg/dL) + infusion 60 mg/kg	Placebo	Haemocomplettan P® CSL Behring, Germany	Fibrinogen concentrate (*n =* 56) vs. placebo(*n =* 55) ^*^Red cells during hospital stay (ml/kg): 29 (24–42) vs. 29 (22–39), ***p=*** **0.36** ^*^FPP volume during hospital stay (mL/kg): 8.8 (0–20.5) vs. 0 (0–20), ***p=*** **0.55** ^*^Plt volume during hospital stay (mL/kg): 0 (0–0) vs. 0 (0–0), ***p=*** **0.85** Total post-operative blood loss (mL/kg): 81 (68–103) vs. 78 (64–97), *p =* 0.16 ICU stay (h.): 20.7 (19.7–21.5) vs. 20.6 (19.8–21.3) Hospital stay (d.): 5 (5–5) vs. 5 (5–5)
**Scoliosis surgery**						
Haas et al. (29)	Single center—Switzerland Staff in PICU blinded	Children (median age 12 years) undergoing scoliosis surgery *N* = 19	Early Fibrinogen replacement FIBTEM MCF <13 mm	Conventional replacement FIBTEM MCF <8 mm	Haemocomplettan P® CSL Behring, Germany (30 mg/kg)^#^	Early (*n =* 10) vs. conventional (*n =* 9) ^*^Total red cells in 24 h. (mL/kg): 0 (0–15.3) vs. 18.4 (0–23.8), ***p=*** **0.21** Total FFP in 24 h. (mL/kg): 0 (0–0) vs. 0 (0–0), *p =* 1.0 Total Plt in 24 h. (mL/kg): 0 (0–0) vs. 0 (0–0), *p =* 1.0
						Calculated blood loss (%): 36.5 (14.9–54.3) vs. 51 (38.5–69.2), *p =* 0.17 ICU stay (d): 1 (1–1) vs. 1 (1–1), *p =* 0.5 Hospital stay (d.): 8 (8–10) vs. 8 (6–13), *p =* 0.57
Chen et al. (30)	Single center–China Blinded	Children (12–18 years) undergoing scoliosis surgery, *N* = 102	Fibrinogen concentrate (30 mg/kg to a maximum dose of 2,000 mg)	Placebo	FIBRORAAS, Shanghai RAAS Blood Products Co, Ltd, Shanghai	Fibrinogen concentrate (*n =* 51) vs. placebo (*n =* 51) ^*^Perioperative blood loss (mL): 885 (755–1,155) vs. 1,035 (818–1,420), ***p=*** **0.041** Perioperative red cells (U): 0 (0–0) vs. 0 (0–0), *p =* 0.34 Perioperative FFP (mL): 0 (0–0) vs. 0(0–0), *p =* 0.29 Hospital stay (d.): 6 (5–7) vs. 7 (5–8), *p =* 0.45

*CPB, Cardiopulmonary bypass; Cryo, Cryoprecipitate; d., days; FC, Fibrinogen concentrate; FFP, fresh frozen plasma; h., hour; ICU, intensive care unit; ITT, intention to treat; MCF, maximum clot firmness; PP, per protocol; Plt, platelets; U, units; UK, United Kingdom; US, United States of America*.

* *(in bold) Primary outcome of study*.

# *Note concurrent coagulation management algorithm included administration of intraoperative FXIII concentrate if FXIII <30%, or between 30 and60% during an episode of bleeding requiring transfusion*.

From the list of ongoing trials identified by the Cochrane systematic review (27), an additional RCT in children (sample size 30) undergoing cardiac surgery has completed recruitment and the results are awaited (35).

### Evidence Translated in Guidelines

Whilst the evidence to support fibrinogen supplementation in children is sparse, guidance regarding fibrinogen supplementation has been included in a number of neonatal and pediatric transfusion guidelines (24, 25, 36–38), as well as some adult-centric guidelines (36, 39–44). See Table 1 for more details. Transfusion guidance provided by national European blood transfusion guidelines differs, since cryoprecipitate is not available and instead, fibrinogen concentrate is used to treat acquired hypofibrinogenemia. There is, however, no consensus European statement or guideline that provides guidance about fibrinogen concentrate indications, thresholds and doses for fibrinogen replacement in neonates and children. Local European guidelines are often difficult to access since they are usually only written in their native language.

The decision to provide fibrinogen replacement to a child, should involve an evaluation a child's condition, the presence of active bleeding, in conjunction with the results of laboratory and hemostatic testing (24, 36). Most guidelines recommend against the transfusion of cryoprecipitate to correct coagulation abnormalities in neonates and children who are not bleeding (24, 25, 38). However, guidance differs between transfusion guidelines with regard to product choice, clinical indications, target fibrinogen levels and doses, reflecting uncertainty, and lack of evidence in these areas. See Table 1 for further details.

### Variability in Practice

It is not surprising, that given the paucity of evidence to support the use of cryoprecipitate and fibrinogen concentration that there is considerable variability in practice and inappropriate use (45, 46).

A single-center retrospective review of 44 critically ill children in the US found that cryoprecipitate was most commonly given in the setting of recent cardiac surgery, DIC, and sepsis (45). More than 60% of transfusions did not meet local institutional indications (45). Many children received cryoprecipitate empirically without a pre-transfusion fibrinogen level and the median dose received was higher than local guidelines suggested (45).

An Australian audit of cryoprecipitate use included 21 cryoprecipitate transfusion episodes for neonates (aged <4 months) and 24 for children (4 months−17 years). A pre-transfusion fibrinogen level and an appropriate indication for transfusion were only present in 57% of neonates and 67% of children (47).

*In summary, there are an increasing number of insufficiently powered RCTs that have evaluated fibrinogen supplementation. RCTs are mainly performed in adults, with only eight small published pediatric RCTs. Due to heterogeneity in clinical settings and indications, their results could not be pooled. There is some evidence in systematic reviews that prophylactic fibrinogen concentrate may result in reduced blood loss and reduced transfusion requirements, compared with inactive comparator. There is no evidence to favor cryoprecipitate or fibrinogen concentrate over one another*.

*Current guidance provided in international and national guidelines for fibrinogen replacement indications, thresholds and dosing are going to be limited by the lack of high quality and well-powered trials. Consensus recommendations or expert opinion statements are likely to be based on the results of a few underpowered trials in specific pediatric patient cohorts*.

## Indications for Fibrinogen Replacement in the Critical Care Setting

### Reduced/Absent or Abnormal Fibrinogen Synthesis

#### Congenital Fibrinogen Disorders

Acute presentations of congenital bleeding disorders are uncommon. Congenital fibrinogen disorders (afibrinogenemia and hypofibrinogenemia) are exceedingly rare, estimated incidence of 1–2 per million people (48). They are classified based on antigenic and functional levels of fibrinogen and include quantitative and qualitative defects (49). Afibrinogenemia is characterized by an undetectable fibrinogen level and results in a significant bleeding phenotype, with both spontaneous bleeding (e.g., umbilical cord, muscle, gingival, and intracranial hemorrhage [ICH]), as well as trauma and surgery-associated bleeding (50, 51). ICH is a major cause of death in these patients (51, 52). Bleeding is typically less severe in hypofibrinogenemia and usually, but not always, correlates with the fibrinogen level (49).

Treatment of congenital fibrinogen disorders will depend on the bleeding phenotype, fibrinogen level and family history. Fibrinogen concentrate is the recommended fibrinogen replacement therapy to treat and prevent bleeding in patients with congenital fibrinogen disorders (53). A fibrinogen level of 100 mg/dL is typically targeted for treatment of minor bleeding, with a higher level of 150 mg/dL targeted for major bleeding, such as ICH (54).

Cryoprecipitate (preferably pathogen-reduced, where available) should only be used as an emergency treatment when fibrinogen concentrate is not accessible. In this context only, a dose of 15–20 mL/kg of pathogen-reduced cryoprecipitate is suggested (55).

*In summary, congenital fibrinogen disorders are rare. The optimal treatment and prevention of bleeding is fibrinogen concentrate*.

#### Liver Disease

The liver plays a key role in the synthesis of multiple hemostatic proteins. Acquired hypofibrinogenemia may be seen when hepatic damage is severe enough to compromise synthetic liver function (56). Liver dysfunction also results in dysfibrinogenemia, with synthesis of an abnormally functioning fibrinogen (56).

In children with severe liver-disease, our understanding of hemostasis is predominantly derived from the adult literature (10). However, this may not be appropriate, given the etiologies of pediatric liver failure differ considerably compared with those seen in adults (57) and hemostasis is age-dependent.

In adults with cirrhosis, low fibrinogen levels (<60 mg/dL) have been highlighted as a predictor for major bleeding (58). Prophylactic fibrinogen replacement has been evaluated in on a RCT conducted in adults undergoing liver transplantation randomized to prophylactic fibrinogen concentrate (dosed to target Fibrinogen of 290 mg/dL) vs. placebo (59). The study found no difference in the study's primary outcome of red cell transfusion requirements (59).

Low quality recommendations or consensus statements are made regarding fibrinogen supplementation in adult liver disease guidelines. Most advise against prophylactic replacement of coagulation factors and that replacement be limited to instances of active bleeding or high-risk invasive procedures (60–63). Some guidelines specify that in the context of active bleeding, a fibrinogen level of >100 mg/dL be maintained (61, 64).

However, this has not been established in pediatric studies. Children with liver failure rarely develop significant spontaneous bleeding (65) indicating that our understanding of the hemostatic equipoise seen in infants and children with advanced liver disease is limited (10). The role of prophylactically correcting laboratory values in children who are not bleeding or undergoing invasive procedures needs to be justified. However, in the context of active bleeding, children with acquired hypofibrinogenemia secondary to liver disease may benefit from fibrinogen replacement (65).

*In summary, the risk of bleeding in children with advanced liver disease and acquired hypofibrinogenemia appears to be less than in adults. Therefore, routine prophylactic fibrinogen supplementation is not advised. There may, however, be a role for correcting hypofibrinogenemia in the context of significant bleeding and advanced liver disease*.

#### L-Asparaginase

L-asparaginase is a key component in pediatric acute lymphoblastic leukemia (ALL) induction chemotherapy protocols (66, 67). As a secondary effect it inhibits the synthesis of hepatic L-asparagine dependent proteins, leading to significant reductions in antithrombin and fibrinogen (68). Hypofibrinogenemia is frequently seen (67), however due to reduced antithrombin levels, thrombotic rather than bleeding complications are encountered (66).

There is a paucity of evidence evaluating fibrinogen replacement in children with acute leukemia and thresholds are largely consensus based and derived from other clinical settings (69). Prophylactic fibrinogen supplementation in this setting is controversial (69–71). Some advocate for therapeutic-only treatment or when there is an increased risk of bleeding due to co-morbidity (70). Others recommend prophylactic cryoprecipitate in all oncology patients with acquired hypofibrinogenemia secondary to medications (72, 73).

*In summary, L-asparaginase is frequently used to treat pediatric ALL and commonly causes acquired hypofibrinogenemia. Current evidence does not point towards an increased bleeding tendency. Therefore, many pediatric ALL protocols do not recommend prophylactic correction with fibrinogen replacement. The role of fibrinogen supplementation in the bleeding child receiving L-asparaginase is unknown*.

### Fibrinogen Loss Exceeding Fibrinogen Production

Massive blood loss may be encountered in children, in the setting of refractory surgical bleeding and trauma (74). Fibrinogen is the first coagulation factor to fall to a critical level of 100 mg/dL and may be encountered after loss or replacement of 1–1.5 blood volumes (75). Additional factors that may contribute to hypofibrinogenemia developing in pediatric trauma and surgical settings include: lower baseline fibrinogen levels in children, hemodilution from crystalloids, or unbalanced hemostatic resuscitation (11), clotting factor consumption, as well as hyperfibrinolysis.

#### Pediatric Cardiac Surgery

Neonates and children undergoing major cardiac surgery are at risk of post-operative bleeding and as a result, commonly receive red cell transfusions (76). Cardiopulmonary bypass (CPB) results in hemodilution, clotting factor consumption, platelet dysfunction, and fibrinolysis (77).

We identified five completed RCTs (*n* = 295) specific to the pediatric cardiac surgical setting (17, 18, 32–34). See Table 2 for further details of these studies.

Two RCTs have compared fibrinogen concentrate with placebo, one as part of a TEG-guided transfusion strategy (33) and the other, as part of a feasibility study evaluating intraoperative ROTEM for screening patients at risk of bleeding (32). A further RCT was identified, comparing fibrinogen concentrate with FFP (34).

Cui et al. randomized 40 children (of which 31 were analyzed) with cyanotic heart disease undergoing cardiac surgery to a TEG-guided transfusion strategy using fibrinogen concentrate (500–1,000 mg) compared to a transfusion guided by clinical experience strategy (without fibrinogen concentrate), with a primary outcome of time to chest wall closure (33). There was no significant difference seen in time to chest wall closure, nor total red cell or platelet usage between study arms. There was, however, statistically lower FFP usage in the fibrinogen concentrate arm, (*p* = 0.006). Unfortunately, the study was not blinded, intention-to-treat principle was not applied, and no information was provided about the median dose, the number or timing of fibrinogen concentrate doses, or the TEG intervention points or any adverse events, limiting its applicability to other settings (33). Use of anti-fibrinolytics was not reported.

Siemens et al. enrolled 111 infants with congenital heart disease undergoing CPB surgery to investigate the feasibility of using intraoperative ROTEM® as a screening tool to predict post-operative bleeding and guide fibrinogen replacement (32). The trial included a monitoring arm of 21 children with a FIBTEM-Maximum Clot Firmness (MCF) >7 mm and an intervention arm (FIBTEM-MCF <6 mm) comprising 60 children randomized to individually-dosed fibrinogen concentrate (target FIBTEM-MCF 8–13 mm) and 30 to placebo (32). Co-primary outcomes were FIBTEM-MCF and fibrinogen levels 5 min post fibrinogen concentrate/placebo administration (32). In addition, the study aimed to review the dosing, safety, and efficacy of fibrinogen concentrate. Whilst FIBTEM-MCF and fibrinogen levels were higher in the fibrinogen concentrate arm, no *p-*values were provided. The study was not powered for efficacy outcomes and the authors did not report *p-*values for any transfusion data. They did however, report significantly lower 24-h mediastinal drain losses in those receiving fibrinogen concentrate (*p* = 0.02). Ten cases of thromboembolism were reported in children who received fibrinogen concentrate compared with two in the placebo arm (32). The study was partially blinded; clinicians and patients were blinded to treatment group. All patients received tranexamic acid.

One RCT was identified where fibrinogen concentrate was compared with FFP (34). Massoumi et al. randomly allocated children undergoing cardiac surgery to receive either FFP (10 ml/kg) or fibrinogen concentrate (70 mg/kg), with a primary outcome of post-operative chest tube drainage. The study reported that chest tube drainage was less in those receiving fibrinogen concentrate (*p* = 0.04). Although not statistically significant due to low numbers, more blood products were given in the FFP-arm. Limitations of the study include the lack of blinding and the method of randomization. Only the first patient was randomized to the fibrinogen treatment arm and subsequent children allocated to FFP or fibrinogen in a 1:1 ratio. Anti-fibrinolytics were not routinely administered.

Two additional RCTs have been conducted comparing cryoprecipitate with fibrinogen concentrate: one in the setting of post-operative bleeding (17) and one as part of a post-CPB protocol (18).

Galas et al. randomized 63 children with diffuse bleeding after CPB and fibrinogen <100 mg/dL to receive 10 mL/kg of cryoprecipitate vs. 60 mg/kg fibrinogen concentrate with a primary outcome of 48-h postoperative blood loss (17). The study found no difference in 48-h blood loss; 320 mL in the fibrinogen concentrate arm vs. 410 mL in those who received cryoprecipitate (*p* = 0.672). There was no statistical difference seen in red cell transfusion rates between study arms; 83.3% of the fibrinogen concentrate arm received a red cell transfusion compared with 97% in the cryoprecipitate arm (*p* = 0.094) (17). Both fibrinogen concentrate, and cryoprecipitate improved fibrinogen levels post intervention. However, there was no difference seen in fibrinogen levels or FIBTEM-MCF between treatment arms and no difference seen for other secondary outcomes (17). Limitations of the study include the lack of blinding of study personnel to treatment allocation. Other considerations are that the cryoprecipitate arm included a higher proportion of neonates and a longer period of cardiopulmonary bypass. Also, more than 40% of patients in both the cryoprecipitate and fibrinogen study groups received additional doses of cryoprecipitate within the first seven post-operative days (17). All patients received ε-aminocaproic acid.

Downey et al. included 59 infants undergoing cardiac surgery randomized to receive two units of cryoprecipitate or fibrinogen concentrate (dosed to target Fibrinogen of 300 mg/dL) as part of a post-CPB hemostasis algorithm with a primary outcome of intraoperative transfusions (18). There was no difference in the red cell, FFP or platelet components received between treatment groups, but the fibrinogen concentrate arm received 1.79 less total blood products compared with the cryoprecipitate arm (intention to treat analysis), likely reflecting the intervention of two units cryoprecipitate (18). Both fibrinogen concentrate, and cryoprecipitate improved fibrinogen levels post intervention. In the per protocol analysis, immediately after administration of fibrinogen supplementation, the median fibrinogen level in the fibrinogen concentrate arm was higher (314.5 mg/dL [IQR: 296.5–342.5]) than the cryoprecipitate arm (276.5 mg/dL [IQR: 192.2–323.5]) (*p* = 0.025). There was also a narrower interquartile range in the fibrinogen concentrate arm, reflecting less variability in the fibrinogen level achieved. A consideration must be the dosing strategy, since the fibrinogen concentrate arm had their dose calculated based on weight, whilst the cryoprecipitate group were administered a standardized dose. The dose of two units cryoprecipitate was based on the author's previous experience and was expected to achieve a post-infusion fibrinogen level of 345 mg. In the study, this dose did not result in the desired level, in is unclear if the reflects product fibrinogen variability or insufficient dosing for weight. There was no difference seen for any secondary outcomes or adverse events. Limitations of the study are that it was not blinded, it was conducted over two centers and there were differences between the two centers with respect to their cardio-pulmonary bypass protocols, the use of four factor prothrombin complex concentrate and the type of antifibrinolytics used. One center used tranexamic acid and the other ε-aminocaproic acid for neonates and those undergoing a re-do sternotomy (18).

An important RCT from the adult cardiac setting included 735 adults with post-operative bleeding and hypofibrinogenemia randomized to 10 units of cryoprecipitate or 4 g of fibrinogen concentrate, with a primary outcome of 24-h cumulative blood products after CPB (78). No difference was seen in the study's primary outcome and no difference was found for secondary transfusion outcomes, mortality or other relevant clinical outcomes (78). It is important to note that the fibrinogen concentrate used (Fibryga®) has much higher levels of FXIII than other fibrinogen concentrates (79).

These studies do not infer superiority of either cryoprecipitate or fibrinogen concentrate but suggest that fibrinogen concentrate may be an alternate, safe, and effective fibrinogen replacement therapy to cryoprecipitate, in the pediatric cardiac surgical setting.

The importance of fibrinogen supplementation in children undergoing cardiac surgery, with evidence of hypofibrinogenemia has been recognized in clinical guidelines. The Network for the Advancement of Patient Blood Management, Haemostasis, and Thrombosis (NATA) pediatric cardiac surgery guidelines recommend that hypofibrinogenemia diagnosed by either Clauss method (<150 mg/dL) or viscoelastic tests (based on an institution-specific algorithm) should be treated with cryoprecipitate or fibrinogen concentrate (37). See Table 1.

#### Pediatric Surgery

Children undergoing cardiothoracic surgery, liver transplantation, craniofacial and neurosurgical procedures, hepatobiliary surgeries, and tumor resection procedures have a high frequency of transfusion (80) and may need PICU admission (81). Craniosynostosis surgery in particular, is frequently associated with coagulopathy and transfusion. Hypofibrinogenemia has been identified as a major risk factor for bleeding in this setting (82).

Our review identified three trials (*n* = 262) evaluating fibrinogen supplementation in the scoliosis and craniosynostosis surgery settings (29–31). See Table 2 for further details.

The timing of preemptive fibrinogen concentrate use has been studied in one pediatric RCT combining infants (median age 10 months) undergoing craniosynostosis surgery and adolescents (median age 12 years) undergoing scoliosis repair (29). Haas et al. randomized 30 children to receive 30 mg/kg fibrinogen concentrate at two predefined FIBTEM cut-offs, a MCF of <8 mm (conventional arm) vs. <13 mm (early substitution arm) with a primary outcome of cumulative volume of red cells received in the first 24 h. Red cell transfusion requirements in the craniosynostosis group were significantly lower in the early fibrinogen replacement group (28 vs. 56 mL/kg, *p* = 0.03). In addition, total blood loss was less in the early fibrinogen replacement group (89.7 vs. 156.9%, *p* = 0.02). There was however, no difference found for red cell transfusion requirements or blood loss in the scoliosis cohort. It is important to note that FXIII replacement was included as part of the study protocol and all subjects received tranexamic acid (29). Specific study limitations were that it was only partially blinded, with only PICU staff blinded to intervention arm and the small sample sizes of the two surgical cohorts. The study ceased early due to difficulties with recruitment with only 57 (instead of 60) subjects randomized. In addition, seven of the 26 randomized scoliosis patients did not meet the FIBTEM trigger for fibrinogen supplementation and could not be included in the analysis (29).

To evaluate the effect of prophylactic administration of fibrinogen concentrate in the craniosynostosis surgery setting, Machotta et al. randomized 114 children to receive fibrinogen concentrate vs. placebo, with a primary outcome of transfusion volume during hospital stay (31). Fibrinogen concentrate (dose to target a fibrinogen of 300 mg/dL [median dose 79 mg/kg]), followed by an infusion of 60 mg/kg during the first hour of surgery (31). Of the 111 children analyzed, fibrinogen concentrations were significantly higher in the fibrinogen treatment arm, but there was no significant difference between the study arms with regard to the transfusion volume, perioperative blood loss, or secondary outcomes. Median volume of red cells received by patients was 29 mL/kg in both the fibrinogen concentrate and placebo arms (*p* = 0.36) (31). This was a double-blinded placebo-controlled trial and the authors attempted to limit confounders, by restricting use of perioperative tranexamic acid, heparin, or non-steroidal anti-inflammatory drugs. They did, however, allow the use of hydroxyethyl starch (HES) which can cause falsely reduced fibrinogen levels (83). Limitations of the study were the slow study recruitment and early termination after 114 out of the planned 120 patients. Three patients were excluded from the final analysis because they received the wrong intervention or no intervention (83).

Chen et al. conducted a blinded RCT, comparing prophylactic fibrinogen concentrate (30 mg/kg) with placebo in 102 adolescents with idiopathic scoliosis undergoing surgery (30). The study's primary outcome was perioperative blood loss, which included intraoperative blood loss and postoperative wound drainage. Fibrinogen concentrate resulted in statistically reduced perioperative blood loss, median reduction of 155 mL (95%CI: 5–320 mL) (*p* = 0.04) compared with the placebo arm. There was no difference found in transfusion rates between study arms. Administration of fibrinogen concentrate resulted in statistically increased fibrinogen levels and MA on the TEG-FF assay. Postoperative fibrinogen levels were inversely correlated with postoperative bleeding (*p* < 0.001) (30). All patients received tranexamic acid and intraoperative cell salvage. The trial stopped prematurely due to a shortage in fibrinogen concentrate, with 102 out of the intended 104 participants recruited (30).

RCTs from both the pediatric cardiac and craniofacial surgery setting suggest that there are different risk factors that influence the need for fibrinogen supplementation. A prospective study looking at predictors of blood loss and red cell transfusion in children undergoing craniosynostosis surgery, found that the type of procedure, the duration of surgery, in addition to post-operative TEG parameters, α-angle, MA, and K-time were all associated with transfusion. Based on these results they developed a TEG-based predictive algorithm for children with critical blood loss (>60 mL/kg) to guide administration of hemostatic products, including fibrinogen concentrate (84).

The 2016 European guideline for the management of severe perioperative bleeding, do not make any recommendation for fibrinogen replacement in pediatric surgery, stating that neither the optimal threshold for initiation of fibrinogen replacement nor the dose required to reach the targeted fibrinogen concentration have been proven by high quality data (41).

In contrast, both the Australian pediatric PBM guidelines and National Institute for Health and Care Excellence (NICE) 2015 guidelines provide guidance around using cryoprecipitate to treat active bleeding in surgical settings when the fibrinogen is <150 mg/dL (24, 39). Neither of these guidelines however, mention FIBTEM or TEG-FF thresholds. See Table 1.

*In summary, hypofibrinogenemia is recognized as a risk factor for perioperative bleeding, in both cardiac and general pediatric surgery. The highest quality evidence for the use of fibrinogen supplementation in children is found in the pediatric cardiac, scoliosis and craniofacial surgery settings. A number of small RCTs (mean number of participants 77, range [31-111]) have been performed. Results from systematic reviews published and RCTs appear to indicate that fibrinogen concentrate may reduce bleeding and reduce the risk of a patient needing a transfusion, compared with no intervention. Unfortunately, current data cannot be pooled due to heterogeneity. Therefore, we can only conclude, that there may be a role for fibrinogen supplementation in young children undergoing high-risk surgical procedures. No superiority of cryoprecipitate compared with fibrinogen concentrate have been demonstrated. Thrombo-embolic events should be considered in children receiving fibrinogen replacement*.

#### Pediatric Trauma

Accidental injuries are the leading cause of death in children and high rates of blunt trauma and traumatic brain injury (TBI) are seen in children compared with adult trauma patients (12, 85, 86). Acute traumatic coagulopathy is commonly encountered in severely injured children prior to the administration of fluids and transfusion (87–90). Prolonged PTs/elevated INRs in trauma patients, including those with TBI appear associated with increased mortality (12, 87, 91). Whilst hypofibrinogenemia has been reported to be an independent predictor of mortality in adult major trauma patients (92, 93), this has not been consistently shown in pediatric studies (12, 87, 90, 94).

In the adult trauma setting, validation studies in the ROTEM® have shown that the FIBTEM CA5 (clot amplitude at 5 min) (95), FIBTEM-MCF and FIBTEM A10 (clot amplitude at 10 min) (96) may be used to predict patients requiring massive transfusion and be used as triggers for fibrinogen supplementation. In the pediatric trauma setting, viscoelastic testing is increasingly used, but studies are needed to establish intervention thresholds (97).

At this moment, there is no high-quality evidence that treatment of hypofibrinogenemia in pediatric trauma patient results in improved patient outcomes. A single case report discusses the successful use of fibrinogen concentrate in a pediatric trauma patient with severe abdominal and pelvic injuries after blunt trauma, without the need for FFP or platelet transfusion (98). However, in the adult combat setting, a retrospective review of 252 trauma patients in Iraq, found that delivery of a higher fibrinogen to red cell ratio was independently associated with improved survival in patients requiring massive transfusion (99).

Nevertheless, pediatric consensus transfusion guidelines do support fibrinogen replacement during critical bleeding and massive blood loss (24, 25). The British Society of Hematology (BSH) advises to target a fibrinogen of >150 mg/dL in massive blood loss and after >40 mL/kg red cells to consider cryoprecipitate transfusion (25). The Australian guidelines support the use of higher targets of 200 mg/dL during critical bleeding, using cryoprecipitate (24). Recent European trauma guidelines do not provide specific recommendations for children, but state that children in general can be managed the same way as adults (100). They recommend one of two initial resuscitation strategies for massive hemorrhage; either FFP and red cells in a ratio of at least 1:2 or fibrinogen concentrate and red cells (100). If there is major bleeding and hypofibrinogenemia (viscoelastic signs of functional fibrinogen deficit or fibrinogen <150 mg/dL) they recommend fibrinogen supplementation with either fibrinogen concentrate or cryoprecipitate (100). However, if fibrinogen concentrate-based management is used, they suggest that FXIII be included in coagulation support algorithm (100). See Table 1.

The Fibrinogen Early in Severe Trauma Study (FEISTY) Junior is an Australian pediatric feasibility RCT comparing the use of early fibrinogen concentrate against cryoprecipitate in children with severe trauma using a ROTEM® based transfusion algorithm (FIBTEM A5 <10 mm as their threshold for intervention) (101). The study's primary outcomes include: time to administration of fibrinogen replacement, feasibility outcomes, and effect on fibrinogen levels (101).

*In summary, coagulopathy including hypofibrinogenemia frequently occurs in pediatric trauma patients. There is limited evidence in the literature supporting or refuting fibrinogen supplementation in the pediatric trauma settings. Both cryoprecipitate and fibrinogen concentrate are widely used to provide fibrinogen supplementation in pediatric trauma patients and form key components in pediatric massive transfusion protocols, with evidence extrapolated from adult trials. More research is needed to identify pediatric categories and triggers in which fibrinogen supplementation is beneficial*.

### Hyperfibrinolysis

#### Disseminated Intravascular Coagulopathy

Disseminated intravascular coagulopathy (DIC) is an acquired, life-threatening condition, resulting in systemic coagulation activation and is associated with both bleeding and thrombosis (102). In children, DIC most commonly occurs secondary to sepsis, but may be seen after major trauma, TBI, malignancy, snakebites, and in vascular malformations (14). The diagnosis of DIC is based on the presence of an underlying causative disorder in conjunction with a combination of laboratory features, including hypofibrinogenemia and fibrinolysis (103). Fibrinogen levels as a single parameter are insensitive in predicting DIC, as they may be elevated as an acute phase reactant and therefore serial testing may be important (104).

The most important aspect of DIC management is timely and appropriate treatment of the underlying condition (103). Transfusion should be reserved for children with active bleeding and should not be given based on laboratory parameters alone (14). The International Society of Thrombosis and Hemostasis (ISTH) recommend fibrinogen replacement only in patients with active bleeding and persistently low fibrinogen levels <150 mg/dL despite treatment with FFP (103). The more recent BSH pediatric transfusion guideline recommends cryoprecipitate be given if the fibrinogen is <100 mg/dL despite FFP or in conjunction with FFP for a very low or rapidly falling fibrinogen (25).

*In summary, DIC is a clinico-pathological diagnosis. The focus of management of DIC should focus primarily on treating the underlying condition. The role of fibrinogen replacement is subject to debate but may be indicated in children with clinically significant bleeding and severe hypofibrinogenemia. The optimal fibrinogen replacement product is unknown*.

#### Extracorporeal Membrane Oxygenation

Extracorporeal membrane oxygenation (ECMO) is increasingly used in neonatal and pediatric critical settings to provide life-saving cardiopulmonary support (3). Neonates and children make up more than half of all ECMO runs reported to the international ELSO registry (105). ECMO circuits comprise artificial and non-endothelial surfaces that, when presented to patient blood, result in fibrinogen adsorption, contact pathway activation, coagulation activation, thrombin generation and fibrinolysis (13, 106). ECMO provokes a significant hemostatic challenge, and as a result both bleeding and thrombotic complications are commonly reported (3, 107, 108). Bleeding in children on ECMO is significantly associated with an increased risk of death (3, 107, 108).

A prospective observational cohort study of 514 pediatric and neonatal ECMO patients identified major bleeding (defined as blood loss requiring transfusion or ICH) in more than 70% of children (3). Whilst fibrinogen levels were monitored on more than 90% of ECMO days, a low fibrinogen was not identified as a risk factor for bleeding (3). In contrast, a small retrospective study of 32 neonates with persistent pulmonary hypertension requiring ECMO reported that low fibrinogen levels were associated with a higher incidence of ICH (109).

It is advised that fibrinogen levels are regular monitored (108, 110, 111), however no RCTs have been performed in this patient cohort looking at fibrinogen thresholds and supplementation and hence there is no consensus on the desired fibrinogen levels needed (110). There is variable practice with respect to fibrinogen supplementation in this cohort. The ELSO guidelines, advise for transfusions of plasma or cryoprecipitate to maintain fibrinogen levels >150 mgd/L in neonates (112) and 250–300 mg/dL in children (111, 112). Whilst other centers report targeting fibrinogen levels >100–150 mg/dL in neonates (108) and fibrinogen levels >200 mg/dL in children (110).

Use of fibrinogen concentrate to treat and prevent bleeding complications is reported in adults on ECMO (113, 114), but not children.

*In summary, major bleeding is commonly seen in pediatric ECMO patients and hypofibrinogenemia may be encountered. Evidence supports regular monitoring, but appropriate triggers for supplementation with either plasma, cryoprecipitate or fibrinogen concentrate are unknown. Considering the high incidence of bleeding in children on ECMO, this area deserves more attention and research*.

#### Thrombolysis in the Pediatric Setting

Venous and arterial thromboembolism are commonly encountered in the pediatric critical care setting (115). Treatment largely comprises targeted anticoagulation with intravenous heparin or low molecular weight heparin (115). Thrombolysis has a role in the treatment of life and organ threatening thrombosis and tPA is the most commonly studied and used (116, 117).

Systemic thrombolysis with tPA results in decreased fibrinogen levels, with many children developing significant hypofibrinogenemia and major bleeding (118). A retrospective study of 79 children treated with systemic tPA for thromboembolism, found that 56 experienced a drop in fibrinogen levels (median decrease 100 mg/dL) and 10 children had fibrinogen levels <100 mg/dL. Overall, bleeding occurred in 54 (68%) children, with 31 (39%) requiring a red cell transfusion. Bleeding appeared to be seen in those who had the largest reductions in their fibrinogen levels following thrombolysis (118).

Due to substantial bleeding risk, pediatric thrombolysis guidelines advise that fibrinogen be maintained >100 mg/dL during thrombolysis and major bleeding with hypofibrinogenemia be treated with cryoprecipitate (116, 117, 119). Use of fibrinogen concentrate is reported in adult stroke patients with severe hypofibrinogenemia following treatment with tPA (120).

*In summary, children receiving systemic tPA are at significant risk of hypofibrinogenemia and bleeding complications. The evidence is too scarce to define a fibrinogen replacement trigger during or shortly after tPA. On the basis of retrospective data, general consensus advises to maintain fibrinogen levels of* >*100mg/dL*

#### Acute Leukemia

Hypofibrinogenemia may be seen in the context of pediatric leukemia associated DIC and is frequently encountered with acute promyelocytic leukemia (APML). APML cells express pro-coagulants including tissue factor and cancer procoagulant, as well as fibrinolytic proteins (plasminogen activators [t-PA and u-PA] and inhibitors [PAI-1] and their receptors [annexin II]) (121). As a result, APML is typically associated with a severe coagulopathy, consistent with a picture of DIC, excess hyperfibrinolysis, and marked hypofibrinogenemia (122). In children, hemorrhage is the main cause of early mortality (123). APML is a pediatric medical emergency necessitating urgent treatment with all-trans-retinoic acid (ATRA). ATRA induces terminal differentiation of malignant promyeloblasts to mature neutrophils (124). This cellular differentiation results in loss of the procoagulant and fibrinolytic properties of the APML cells, with improvement in bleeding symptoms and coagulopathy (121, 123).

There are no trials evaluating fibrinogen replacement triggers in APML. Major clinical trials advise that fibrinogen levels be monitored and treated with transfusions (124). The Pediatric International Consortium for Childhood APL (ICC- APL- 01) trial advises that fibrinogen levels >150 mg/dL be maintained during the first 10 days of induction therapy or until resolution of any coagulopathy, using FFP (125). The Children's Oncology Group (COG) Study AAML0631 trial protocol advises that the fibrinogen be maintained >100 mg/dL (126). The European LeukemiaNet guidelines suggest that following the diagnosis of APML immediate fibrinogen supplementation (FFP, fibrinogen, and or/cryoprecipitate) be implemented to maintain a fibrinogen concentration >100–150 mg/dL and to continue through induction therapy until the coagulopathy resolves (127). Similar guidance is provided in the Canadian Blood Transfusion Guideline (42).

*In summary, APML-associated hypofibrinogenemia carries a high risk of bleeding. The only definitive treatment is urgent treatment with ATRA, but in instances of clinically significant bleeding bridging with fibrinogen supplementation is advised on expert opinion. Most guidelines advise preemptive supplementation when fibrinogen levels fall* <*100mg/dL, but the optimal product is unknown*.

#### Hemophagocytic Lymphohistiocytosis

Hemophagocytic lymphohistiocytosis (HLH) is a rare, life-threatening inflammatory syndrome with a peak incidence in infancy. It results from a dysregulated immune response that leads to pathological hyperactivation of NK cells, T lymphocytes, and macrophages (128). Hypofibrinogenemia (<150 mg/dL) is one of the eight criteria, included in the 2004 HLH diagnostic criteria, where five out of eight criteria are required for diagnosis (129). Diagnostic workup for HLH in a child will include immune and genetic evaluation to measure proteins affects in familial HLH and viral serologies (EBV and CMV) to identify a common viral trigger for HLH (130).

It is not known, why hypofibrinogenemia develops in HLH, proposed mechanisms include: hyperfibrinolysis secondary to DIC, decreased fibrinogen production secondary to hepatic macrophage infiltration, cytokine storm, and hyperfibrinogenolysis (131). A retrospective review of 117 adults with HLH, reported that fibrinogen levels <200 mg/dL were associated with severe bleeding and independently associated with higher mortality (131). On the contrary, retrospective studies looking at predictors for mortality in children with HLH, did not identify hypofibrinogenemia as being a significant factor (132–134). Acute bleeding in the setting of hypofibrinogenemia and HLH may require fibrinogen supplementation (135). In general though, hypofibrinogenemia will only resolve with definitive HLH-treatment with etoposide and dexamethasone (130). Guidance around triggers for prophylactic fibrinogen supplementation in children was not found.

*In summary, hypofibrinogenemia is one of the eight criteria used in making the diagnosis of HLH. Patients with HLH are at risk of bleeding. The only definitive treatment is HLH-treatment with etoposide and dexamethasone. In cases of clinical bleeding and severe hypofibrinogenemia bridging with fibrinogen supplementation may be considered*.

## Neonates and Hypofibrinogenemia

There are many age-dependent differences in fibrinogen and fibrinolysis in neonates, compared with older children. Regardless of the relative immaturity in the neonatal hemostatic system, in general neonates appear to have effective and balanced coagulation, and do not tend to bleed spontaneously (136).

In the critical care setting (NICU), neonates have different etiologies for acquired hypofibrinogenemia compared with older children. DIC in the neonate, most commonly occurs in the setting of perinatal asphyxia, but can be due to sepsis and perinatal acquired infections, respiratory distress syndrome and meconium aspiration (14, 137, 138). Severe perinatal asphyxia can cause significant hypoxic brain injury and multi-organ failure, including hepatic damage (139, 140). Hypoxic hepatic damage can lead to reduced production of coagulation factors and hypofibrinogenemia (140).

Rare, but potentially life-threatening causes of acquired hypofibrinogenemia in neonates include purpura fulminans due to congenital deficiency of protein C or protein S (141) and Kasabach-Merritt phenomenon (an acute consumptive coagulopathy specifically associated with two vascular tumors) (142). In Kasabach-Merritt phenomenon, a neonate may present with a rapidly growing tumor, with thrombocytopenia and severe hypofibrinogenemia, due to platelet sequestration, and fibrinogen consumption (142). There is a high risk of bleeding and management involves surgical or medical treatment of the tumor. If fibrinogen levels are <100 mg/dL then FFP or cryoprecipitate is recommended, especially in the presence of bleeding (143).

The Italian neonatal transfusion guidelines provide definitions and guidance for fibrinogen replacement in neonates. They recommend observation rather than treatment in neonates with hypofibrinogenemia and no bleeding. But when active bleeding is present or a neonate is about to undergo an invasive procedure and the fibrinogen level is below the lower limit for gestational age, they recommend 5–10 mL/kg of cryoprecipitate (38).

*In summary, neonates tend to have lower fibrinogen levels without an increased bleeding risk. Therefore, most guidelines do not advise to correct asymptomatic hypofibrinogenemia. When an acquired, severe hypofibrinogenemia occurs and there is a high risk of bleeding, fibrinogen supplementation may be considered. The optimal fibrinogen replacement product in neonates is unknown*.

## Current Knowledge Gaps, Controversies, and Areas for Research

Although prophylactic and therapeutic use of cryoprecipitate and fibrinogen concentrate in both congenital and acquired hypofibrinogenemia has been widely adopted in a variety of pediatric critical care situations, there remain many uncertainties and controversies regarding fibrinogen replacement. Similar sentiments are echoed in the current adult literature (144–146).

The evidence base to support fibrinogen replacement in children is sparse or extrapolated from (also sparse) adult studies. We can only emphasize the need for more, well-designed, and sufficiently powered clinical trials in children. Considering the multiple national and international guidelines that exist about the use of fibrinogen in children, and the lack of consensus between guidelines, we see a medical need for international collaboration between guideline-writing groups.

There are number of questions that still need to be answered and may be proposed as areas for future research in neonates, including those preterm, infants, children, and adolescents with regard to clinical indications for fibrinogen supplementation.

Fibrinogen thresholds:

- What is the relationship between fibrinogen and bleeding in neonates and in children?
- What is the relationship between fibrinogen and bleeding in different clinical settings e.g., massive hemorrhage, trauma, CPB, major surgical bleeding, ECMO, liver disease, DIC, and hyperfibrinolysis?
- What are the clinical indications for prophylactic fibrinogen replacement in neonates and children?
- What are the clinical indications for therapeutic fibrinogen replacement in children?
- What is the optimal fibrinogen level for neonates and children?
- undergoing high-risk surgical or invasive procedures?
- presenting with massive blood loss due to severe trauma?
- with other critical care conditions?

Prediction of hypofibrinogenemia-related bleeding and optimal treatment in children:

- How do we predict which neonates and children will bleed during or following surgery, and who are most likely to benefit from fibrinogen supplementation?
- How do we predict which neonates and children are most likely to benefit from fibrinogen supplement following trauma with massive blood loss or TBI?
- How do we predict which neonates and children are most likely to benefit from fibrinogen supplement in the context of sepsis, DIC, leukemia, and vascular tumors?
- Which fibrinogen product is the best choice in the different pediatric critical care clinical settings?

Clinical trials and guidelines:

- How do we increase the quality of clinical trials studying transfusion medicine in neonates and children?
- Can we come to an international consensus when there is a lack of evidence to reduce the variability of indications and triggers seen between national and international guidelines?

## Conclusions

Critically ill children frequently experience bleeding events and hypofibrinogenemia is implicated in adult and pediatric settings as an important risk factor for bleeding. There remains considerable uncertainty in children of all ages around optimal fibrinogen levels and the best fibrinogen replacement strategies. Cryoprecipitate and fibrinogen concentrate are both given to prevent and treat bleeding due to hypofibrinogenemia, in spite of a sparse evidence base. Neonates and children continue to be under-represented or underpowered in clinical trials. Further evidence and RCTs in pediatric transfusion medicine are needed, so that, rather than extrapolate from adult studies or base practice on experience, best and evidence-based practice is delivered.

## Author Contributions

GC literature review and writing and editing article. EH reviewing content and editing article. All authors contributed to the article and approved the submitted version.

## Conflict of Interest

EH was an author of one of the RCTs discussed in this article. The remaining author declares that the research was conducted in the absence of any commercial or financial relationships that could be construed as a potential conflict of interest.
